# Spatial Biomarker Deep Learning Model Predicts Response to PI3K Inhibition in Head and Neck Cancer

**DOI:** 10.3390/cancers18121887

**Published:** 2026-06-10

**Authors:** Antoine Desilets, Minh Tri Le, Catalina Moreno, Justin Lucas, Alexandre Pellan Cheng, Orit Matcovitch-Natan, Amit Bart, Avi Laniado, Meir Azulay, Ettai Markovits, Jennifer Kaplan Kerner, Amit Gutwillig, Hadar Yehezkeli, Lisa F. Licitra, Sunny Lu, Kevin Dreyer, Ying Pan, Nanhai He, Archie Tse, Sandrine Faivre, Denis Soulières

**Affiliations:** 1 Hematology-Oncology Service, Department of Medicine, Centre Hospitalier de l’Université de Montréal (CHUM), Montreal, QC H2X 0A9, Canada; antoine.desilets.med@ssss.gouv.qc.ca; 2 Cancer Axis, Centre de Recherche du CHUM (CRCHUM), Montreal, QC H2X 0A9, Canada; minh.tri.le.med@ssss.gouv.qc.ca (M.T.L.); catalina.moreno@umontreal.ca (C.M.); alexandre.pellan-cheng@etsmtl.ca (A.P.C.); 3 Department of Pathology, Centre Hospitalier de l’Université de Montréal (CHUM), Montreal, QC H2X 0A9, Canada; 4 Adlai Nortye, North Brunswick, NJ 08902, USA; lucasjp88@gmail.com (J.L.); xuyang.lu@adlainortye.com (S.L.); kevin.dreyer@adlainortye.com (K.D.); ying.pan@adlainortye.com (Y.P.); nathan.he@adlainortye.com (N.H.); archie.tse@adlainortye.com (A.T.); 5 Department of Systems Engineering, École de Technologie Supérieure, Montreal, QC H3C 1K3, Canada; 6 Nucleai, Tel Aviv 6744840, Israel; oritmat@gmail.com (O.M.-N.); amitbart@gmail.com (A.B.); laniado89@gmail.com (A.L.); meir.azulay@nucleai.ai (M.A.); ettai.markovits@nucleai.ai (E.M.); jennifer.k.kerner@gmail.com (J.K.K.); agutwil@gmail.com (A.G.); hadar1903@gmail.com (H.Y.); 7 Head and Neck Cancer Medical Oncology 3 Unit, Fondazione IRCCS Istituto Nazionale dei Tumori, Department of Oncology and Hemato-Oncology, University of Milan, 20122 Milan, Italy; lisa.licitra@istitutotumori.mi.it; 8 Hôpital St-Louis, 75010 Paris, France; sandrine.faivre@aphp.fr

**Keywords:** buparlisib, squamous cell carcinoma, head and neck cancer, digital pathology, spatial biomarkers, H&E, artificial intelligence, BERIL-1

## Abstract

**Background**: Buparlisib, combined with paclitaxel, improved survival in BERIL-1 trial patients with recurrent/metastatic head and neck squamous cell carcinoma (R/M HNSCC). However, predictive biomarkers of benefit remain undefined. **Objective**: To evaluate whether spatial biomarkers extracted from hematoxylin and eosin (H&E) slides using artificial intelligence (AI) can predict overall survival benefit from buparlisib. **Methods**: Whole-slide H&E images from BERIL-1 trial patients were analyzed using a deep learning model trained to segment tissue compartments and classify cell phenotypes. Three predefined spatial features were evaluated: tumor-infiltrating lymphocyte density, tumor microenvironment heterogeneity, and granulocyte fraction in the tumor invasive margin. Cox proportional hazards model assessed biomarker-treatment interactions. **Results**: Of 158 trial participants, 144 had available slides. High tumor-infiltrating lymphocyte density (>10%) was associated with significantly improved overall survival with buparlisib versus placebo (HR, 0.25 (95% CI, 0.01–0.64; *p* = 0.002)), as were high tumor microenvironment heterogeneity (HR, 0.47 (95% CI, 0.27–0.80; *p* = 0.005)) and granulocyte enrichment in the tumor invasive margin (HR, 0.51 (95% CI, 0.30–0.88; *p* = 0.01)); within-arm proximity analysis showed higher granulocyte–tumor-cell proximity correlated with improved overall survival on buparlisib (HR, 0.32 (95% CI, 0.18–0.58; *p* < 0.001)). AI-derived spatial metrics outperformed CD3 immunohistochemistry. Among oropharyngeal tumors, HPV-positive cases were more frequent in patients with high tumor-infiltrating lymphocytes. **Conclusions**: AI-extracted spatial features from H&E slides were associated with overall survival benefit from buparlisib in R/M HNSCC. These scalable biomarkers support image-based patient selection strategies and are being prospectively evaluated in the BURAN phase 3 trial.

## Simple Summary

Currently, there is no reliable way to identify which patients with advanced head and neck cancer are most likely to benefit from treatment with buparlisib, a PI3K inhibitor, making personalized therapy challenging. The aim of our study was to explore whether routine tissue slides can help identify spatial features that predict improved survival with buparlisib. Using an artificial intelligence-based digital pathology model, we examined immune cell infiltration, cellular diversity within the tumor microenvironment, and granulocyte enrichment as predictors of improved overall survival. We also found that these features provided stronger predictive value than conventional immunohistochemistry. The results suggest that computational analysis of tissue slides could offer scalable, cost-effective biomarkers, offering clinicians a new strategy for selecting patients who are more likely to benefit from PI3K inhibitor therapy.

## 1. Introduction

Recurrent or metastatic (R/M) head and neck squamous cell carcinoma (HNSCC) is a highly aggressive malignancy with poor survival outcomes. Despite advances with the advent of immune checkpoint inhibitors (ICI) [1,2] and targeted agents [3,4,5], most patients eventually experience disease progression, underscoring the need for more effective and personalized treatment strategies. Among emerging approaches, phosphoinositide 3-kinase (PI3K) inhibitors have shown promise [6,7]; however, clinical benefit has been heterogeneous across cohorts partly due to the absence of validated predictors of response [8,9,10].

The BERIL-1 clinical trial demonstrated benefits in objective response rate (ORR), progression-free survival (PFS), and overall survival (OS) with the addition of buparlisib, a pan-class I PI3K inhibitor, to paclitaxel in patients with R/M HNSCC who had previously progressed on platinum-based chemotherapy [7]. Translational analyses identified several molecular and immunologic features associated with improved response, including *PIK3CA* and *TP53* mutations, high tumor mutational burden (TMB), and elevated baseline immune infiltration, as measured by CD8+ T cells and tumor-infiltrating lymphocytes (TIL) [8,9]. However, these conventional biomarkers—typically applied using binary predefined thresholds—fail to capture the spatial architecture and complexity of the tumor microenvironment (TME) [11], now recognized as a key modulator of therapeutic response.

Conventional diagnostic pathology approaches, such as immunohistochemistry (IHC) and bulk DNA or RNA sequencing via next-generation sequencing (NGS), offer limited insight into the spatial heterogeneity and cellular architecture of the TME. IHC provides low spatial resolution and is subject to interpretive variability, while NGS is costly, complex, and not universally accessible. In contrast, hematoxylin and eosin (H&E) staining is inexpensive, routinely performed, and widely available, yet remains underutilized as a quantitative biomarker due to the historical lack of tools for extracting interpretable spatial features. Recent advances in artificial intelligence (AI)-driven digital pathology now enable whole-slide H&E analysis to identify clinically meaningful patterns—such as immune infiltration, cellular heterogeneity, and stromal organization—with high spatial resolution, offering critical context for understanding the TME contexture that may influence drug susceptibility and resistance [12,13,14].

In this study, we applied a deep learning pipeline to digitized H&E slides from the BERIL-1 trial to evaluate the prognostic and predictive value of spatial biomarkers. We hypothesized that AI-based spatial annotation of H&E images could identify tumor microenvironmental features—such as TIL density, TME heterogeneity, and granulocyte infiltration—that correlate with response to buparlisib. These features have biological plausibility, as PI3K signaling regulates immune evasion, stromal remodeling, and myeloid cell recruitment [15,16], while chemotherapy can further modulate immune–stromal interactions [17,18,19]. Thus, immunological and spatial features of the TME may directly influence sensitivity to combined PI3K inhibition and paclitaxel. Our goal was to explore whether digital H&E analysis could provide a cost-effective, scalable tool to refine patient selection in future PI3K inhibitor trials. To our knowledge, this represents one of the first studies evaluating AI-derived spatial biomarkers from routine H&E slides as predictors of response to PI3K inhibition in R/M HNSCC within a randomized clinical trial dataset.

## 2. Materials and Methods

### 2.1. Study Design

This retrospective exploratory study analyzed archival H&E-stained tumor slides from patients enrolled in the BERIL-1 trial, a multicenter, randomized, double-blind, placebo-controlled phase II study in adults with histologically or cytologically confirmed R/M HNSCC who had progressed following platinum-based chemotherapy. Patients were randomized to receive intravenous paclitaxel (80 mg/m^2^ on days 1, 8, 15, and 22 of each 28-day cycle) in combination with either daily oral buparlisib (100 mg) or placebo. The primary endpoint was PFS, with OS and ORR as secondary endpoints. Radiological responses were assessed by Response Evaluation Criteria in Solid Tumors (RECIST) version 1.1. Tumor samples were obtained prior to treatment initiation and originated from either primary or metastatic sites.

### 2.2. Tumor Samples and Image Digitization

Formalin-fixed, paraffin-embedded (FFPE) sections from tumor samples at baseline were evaluated to ensure sufficient tumor content and staining quality. Slides were scanned at 40× magnification using a high-resolution whole-slide imaging (WSI) system. The digitized images underwent a standardized quality control review for staining consistency, cellular integrity, and completeness of tumor tissue architecture. Only slides with sufficient tumor content, intact tissue morphology, and acceptable staining quality were digitized for analysis. Slides that failed QC were excluded due to issues such as absence of tumor cells (e.g., slides containing only blood or benign tissue) or poor technical quality (e.g., degraded or poorly stained tissue). Full QC details, including exclusion categories, are provided in Supplemental Table S1. Conventional TIL IHC scoring was performed using CD3 immunostaining on a single whole-tissue section, with lymphocyte density assessed across intratumoral and stromal compartments by visual estimation. Human papillomavirus (HPV) status was assessed in all patients via p16 IHC on FFPE tumor samples, regardless of primary tumor site, and correlated with TIL infiltration. This curated dataset enabled the generation of spatially annotated maps of the tumor and immune microenvironment, forming the basis for subsequent correlation with clinical endpoints.

### 2.3. Deep Learning Model Development Using Digital Pathology

Digitized WSI were analyzed using a deep learning-based digital pathology platform designed to extract spatially resolved features of the TME at a single-cell detection and classification resolution. Rather than performing true instance segmentation to delineate individual cell boundaries, the model identifies and classifies individual cells based on deep learning segmentation and centroid approximation. The final output for each cell comprises its exact x, y coordinates and assigned class. The pipeline consisted primarily of U-Net-based convolutional neural network (CNN) architectures optimized for tissue segmentation and single-cell classification tasks. The two core modules are: (1) a spatial segmentation model to delineate histologic compartments, and (2) a single-cell classification model to characterize the phenotypic composition of the TME, encompassing principal immune and stromal cell types. The models leveraged pretrained CNNs (primarily U-Net architecture) developed on large multi-tumor datasets and were further refined using cohort-specific annotations from the BERIL-1 trial samples. The U-Net variant used was 576 × 576 × 3 RGB input, base channel width 32, encoder/decoder channel multipliers [1,2,4,8,16], and six output channels. The H&E images are handled as RGB throughout; they are not converted to grayscale. Annotated data were partitioned into training, validation, and test datasets during model development. The dataset partitioning was performed on a slide level. A total of 19 slides were designated as a test set (which was not used in the training process), 19 slides for validation, and the rest were part of the training set. The training set was used to change the model parameters, while the validation set was used to choose the best checkpoint. The test set was used for final evaluation. Several iterations were performed; for each time, additional annotations were added.

Preprocessing included automated tissue segmentation, artifact detection (e.g., folds, ink, and out-of-focus regions), and resolution standardization. During training, several augmentations were used, including rotation, horizontal flipping, cropping, cutout, and color augmentation that was used to simulate staining variability in H&E images, enhancing the model’s robustness and reducing sensitivity to color distribution shifts. To ensure optimal feature extraction and mitigate computational constraints, slides were resampled to 0.5 µm/pixel for cell detection and classification and 1.25 µm/pixel for area segmentation (tumor, stroma, etc.). Image tiles of 576 pixels with 480-pixel overlap (that provide additional augmentation to the CNN due to the overlap) were utilized during training to provide sufficient contextual information while preserving fine morphological details. During inference, overlapping tiles are processed by the network and only the context-trimmed central region is stitched back into the slide-level prediction. The trusted core is 576 − 2 × 192 = 192 pixels.

**Figure 1:**
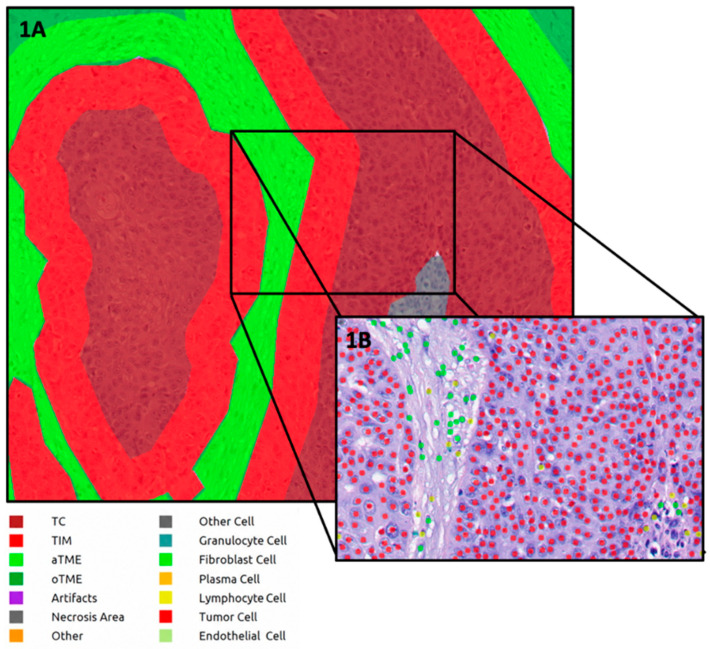
Spatial annotation of the tumor microenvironment and single-cell phenotypes in head and neck cancer. *Legend:* (**A**). Representative H&E-stained whole-slide image processed with automated spatial segmentation. Tumor and stromal regions are delineated based on proximity to the tumor–stroma interface. (**B**). High-resolution region showing overlay of cell-type annotations derived from a deep learning classifier. Individual cells are labeled by phenotype, including tumor cells (red), lymphocytes (green), fibroblasts (yellow), endothelial cells (blue), and others. Color legend indicating spatial compartments (left row) and single-cell phenotypes (right row).

The spatial segmentation model partitioned the tissue into tumor center (TC), tumor invasive margin (TIM), adjacent TME (aTME), and outer TME (oTME), using a predefined 60 µm inner and outer boundary relative to the tumor–stroma interface. These regions were color-coded and overlaid on the H&E slides to enable interpretable visualization of the TME structure (Figure 1A). Cell-type annotations derived from the classification model were rendered at the single-cell resolution and superimposed on representative high-resolution zones to visualize tumor cells, lymphocytes, plasma cells, fibroblasts, endothelial cells, and granulocytes (Figure 1B). Confusion matrices were generated for each model to derive class-specific performance metrics (Supplemental Figure S1).

### 2.4. Evaluation of Single-Cell Classification and Spatial Segmentation Models

The single-cell classification model applied deep CNN trained on expert-labeled data to assign phenotypic identities to individual cells. Targeted cell types included tumor cells, lymphocytes, fibroblasts, plasma cells, granulocytes, and endothelial cells. Annotations were performed by multiple trained annotators with backgrounds in life sciences or medicine and underwent secondary review procedures under supervision of a board-certified pathologist (JKK). Training, validation, and test datasets were separated during model development. To assess the spatial segmentation model, we defined ‘accuracy’ as the per-class pixel accuracy, defined as the number of correctly segmented pixels divided by the total number of pixels of that class in the ground truth. Computed on fully annotated regions with a full confusion matrix, this ensures that over-segmentation of one class necessarily degrades performance in others. Although not fully blinded, comparisons were repeated across multiple runs, and a final holistic evaluation was performed to ensure accuracy across whole-slide images. In parallel, the spatial segmentation model was assessed across five distinct histological regions—tumor, stroma, necrosis, nerve, and other non-neoplastic tissue—by computing concordance between predicted and reference labels at the region level. Only cells with well-defined morphology were included in the ground truth dataset to ensure robust assessment. All performance metrics were independently reviewed and validated by a board-certified pathologist (JKK) to ensure interpretative consistency and accuracy.

### 2.5. Spatial Biomarker Derivation and Quantification

Three spatial biomarkers were prospectively defined and extracted from annotated H&E slides for each patient. These were selected from a larger pool of spatial features (~3000 calculated) based on predefined biological and clinical rationale: (1) the density of TIL within the tumor area—defined as the TC and TIM, excluding stromal regions; (2) cellular heterogeneity of the TME, quantified using the Shannon entropy index across all classified cell phenotypes—a validated non-parametric index of ecological diversity; and (3) the proportion of granulocytes within the TIM, representative of localized innate immune infiltration. Spatial features were computed using AI-based single-cell classification and region-specific overlays. TME heterogeneity and granulocyte fraction were each dichotomized as high or low according to the median value across the cohort. Median-based dichotomization was selected for exploratory analyses in the absence of validated clinical thresholds for PI3K inhibitor response. For TIL, a pre-specified threshold of 10% was used based on prior biological and clinical rationale [8,9]. All biomarker metrics were derived independently of clinical outcomes and later evaluated for association with OS in exploratory analyses. In a secondary proximity analysis, we quantified a granulocyte–tumor-cell proximity metric, defined as the proportion of granulocytes located within 50 µm of tumor cells in the tumor area (TC + TIM) using the same region overlays; this metric was used for within-arm and overall prognostic associations.

### 2.6. Statistical Analysis

The primary clinical endpoint for this retrospective analysis was OS, defined as the time from randomization to death from any cause. OS distributions were estimated using the Kaplan–Meier method and compared between the treatment arms within each of the biomarker-defined subgroups using log-rank tests. Cox proportional hazards models were used to estimate hazard ratios (HR) between the treatment arms and corresponding 95% confidence intervals (CI) within each biomarker-defined subgroup. All biomarker analyses were exploratory; two-sided *p*-values < 0.05 were considered nominally significant without adjustment for multiplicity. Given the exploratory nature of this analysis, no adjustments were made for multiple comparisons. The evaluated biomarkers were prospectively selected from a larger pool of computed spatial features based on predefined biological and clinical rationale. All analyses were conducted in R version 4.2.2 (R Foundation for Statistical Computing, Vienna, Austria). Primary analyses focused on predictive effects, estimating the treatment effect of buparlisib versus placebo within each biomarker-defined subgroup (high/low). Prognostic, within-arm high-versus-low comparisons were performed only as secondary analyses (e.g., the granulocyte–tumor-cell proximity metric).

## 3. Results

### 3.1. Survival Outcomes and Biomarker Landscape of the BERIL-1 Trial

Treatment with buparlisib in the BERIL-1 trial was associated with improved survival outcomes compared to placebo, including a median PFS of 4.6 versus 3.5 months (HR, 0.65 (95% CI, 0.45–0.95; *p* = 0.01)) and a median OS of 10.4 versus 6.5 months (HR, 0.72 (95% CI, 0.49–1.04; *p* = 0.04)). These efficacy results reflect the statistical approach of the original BERIL-1 trial, which used one-sided testing for primary and secondary endpoints. The ORR was also higher in the buparlisib arm (39% versus 14%; nominal one-sided *p* < 0.001), supporting the clinical benefit of PI3K inhibition in this population [7]. Prior translational analyses demonstrated a high frequency of oncogenic alterations—most commonly in *TP53*, *NOTCH1*, and *PIK3CA*. While genomic- and IHC-based immune biomarkers, such as TIL and CD8+ cell infiltration, were broadly prevalent, they did not fully explain differential treatment benefits, prompting further evaluation of spatial biomarkers using digital pathology [8,9]. Unless otherwise specified, *p*-values for biomarker-defined subgroup analyses are reported as two-sided.

**Figure 2:**
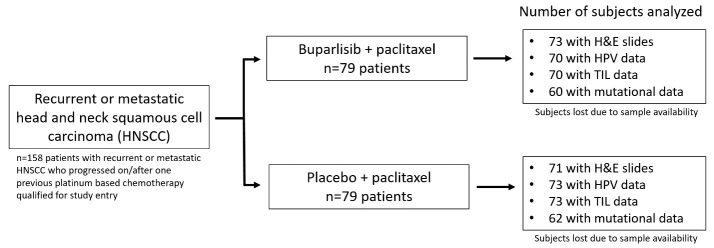
BERIL-1 trial design and translational analysis sample size. *Legend:* Overview of the BERIL-1 phase II clinical trial, which enrolled *n* = 158 patients with recurrent or metastatic HNSCC progressing after prior platinum-based chemotherapy. Patients were randomized to receive either buparlisib plus paclitaxel or placebo plus paclitaxel (*n* = 79 per arm). Translational analyses were conducted on available tissue samples. In the buparlisib arm, 73 patients had H&E slides for digital pathology analysis, 70 patients had HPV and TIL data, and 60 had mutational data from targeted DNA NGS. In the placebo arm, 71 patients had H&E slides for digital pathology analysis, 73 had HPV data, 73 had TIL data, and 62 had mutational data. A total of 76 patients from each arm were included in the H&E-based digital pathology analysis based on tissue availability. *Abbreviations*: HNSCC: head and neck squamous cell carcinoma; H&E: hematoxylin and eosin; HPV: human papillomavirus; TIL: tumor-infiltrating lymphocytes; NGS: next-generation sequencing.

Among the 158 patients enrolled, 144 had available baseline FFPE tumor samples suitable for digital pathology analysis, enabling spatial biomarker extraction. These included 73 patients from the buparlisib plus paclitaxel arm and 71 from the paclitaxel plus placebo arm (Figure 2). Baseline characteristics of patients with evaluable FFPE samples for H&E analysis are presented in Supplemental Table S2.

### 3.2. Deep Learning Model Performance for Tissue and Cell Classification

**Figure 3:**
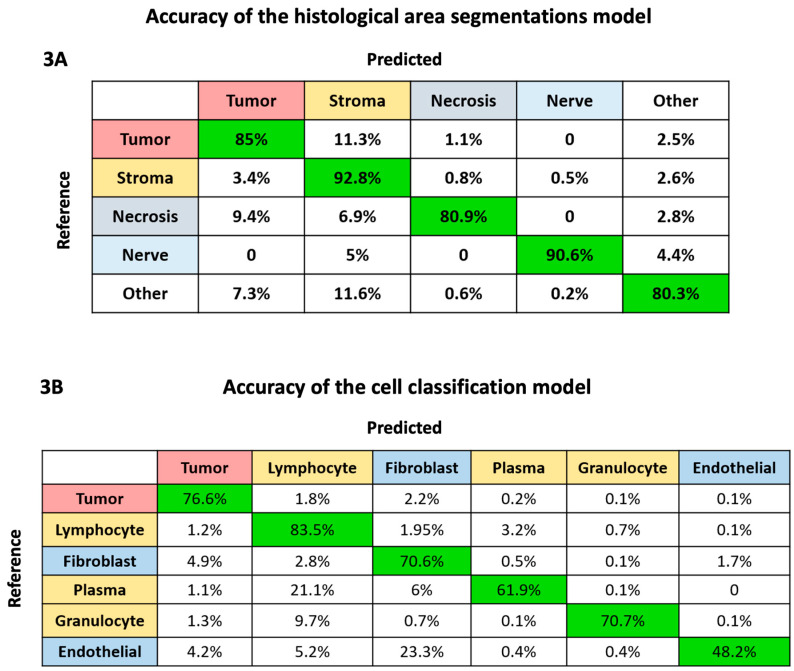
Deep learning performance for segmentation and single-cell classification in head and neck squamous cell carcinoma. *Legend:* (**A**). Area segmentation model performance showing percent agreement between predicted and reference labels for five histological regions. (**B**). Cell classification model performance with percent agreement across seven cell types. Correct classifications are highlighted in green.

To support spatial biomarker extraction, we first evaluated the performance of the deep learning models for histologic region segmentation and single-cell classification. The histological area segmentation model showed strong concordance with expert annotations, with accuracy rates of 85.0% for tumor, 92.8% for stroma, 80.9% for necrosis, and 90.6% for nerve regions (Figure 3A). The area segmentation accuracy is detailed in Section 2.4. The cell classification model accurately identified major stromal and immune phenotypes, achieving the highest agreement for lymphocytes (83.5%), tumor cells (76.6%), fibroblasts (70.6%), and plasma cells (61.9%) (Figure 3B). Performance for granulocytes was moderate (70.7%), while endothelial cell classification was more limited (48.2%). All predictions were reviewed for biological plausibility and integrated into spatially contextualized cell maps, which served as the basis for subsequent biomarker extraction across TME compartments. While plasma cell misclassification with lymphocytes and moderate granulocyte accuracy were noted, these predictions were considered sufficient for exploratory analyses, though granulocyte detection from H&E will require additional validation in future studies.

### 3.3. Quantitative Evaluation of Tumor-Infiltrating Lymphocytes Using H&E Versus IHC

**Figure 4:**
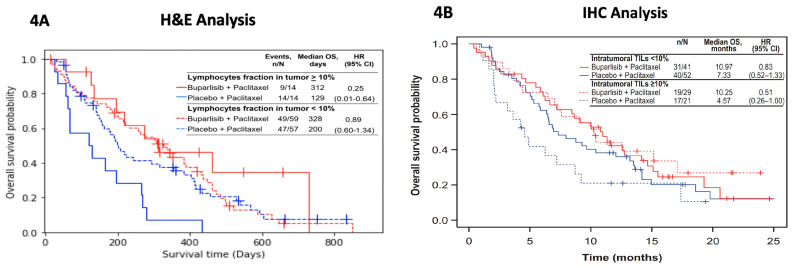
Tumor-infiltrating lymphocytes predict buparlisib response: comparative analysis using hematoxylin and eosin quantification and immunohistochemistry scoring. *Legend:* (**A**). H&E-based lymphocyte fraction analysis: Kaplan–Meier curves of OS stratified by lymphocyte fraction in tumor regions as quantified from H&E-stained slides. (**B**). IHC-based TIL analysis: Kaplan–Meier curves of OS based on IHC-assessed intratumoral TIL.

The prognostic and predictive relevance of TIL was assessed using AI-based spatial quantification of H&E images compared to conventional IHC scoring. Among patients with high TIL density (≥10%), a significant treatment effect of buparlisib versus placebo was observed in patients with high TIL (HR, 0.25 (95% CI, 0.01–0.64, *p* = 0.002)), whereas no between-arm difference was seen in patients with <10% TIL (HR, 0.89 (95% CI, 0.60–1.34, *p* = 0.59)) (Figure 4A). Exploratorily, within the placebo arm, OS trended longer in patients with <10% TIL than in those with ≥10% TIL, whereas no clear within-arm difference was seen in the buparlisib arm; these within-arm high-versus-low comparisons were descriptive and not powered for inference. In contrast, IHC-based TIL scoring yielded a weaker association (HR, 0.51 in the ≥10% group (95% CI, 0.26–1.00, *p* = 0.05); HR, 0.83 in the <10% group (95% CI, 0.52–1.33, *p* = 0.44)) (Figure 4B). Both H&E- and IHC-based TIL analyses suggest that high baseline lymphocytic infiltration in the tumor microenvironment may serve as a prognostic and potentially predictive biomarker for benefit from buparlisib in combination with paclitaxel. H&E-based spatial annotation demonstrated stronger hazard ratios and clearer OS separation than traditional IHC scoring. In a secondary analysis of patients with baseline tumor samples evaluable for HPV status by p16 IHC (*n* = 70 buparlisib; *n* = 73 placebo; see Figure 2), the proportion of HPV-positive tumors was higher among cases with ≥10% TIL than among those with <10% TIL (Supplemental Figure S2), irrespective of treatment arm or primary site.

### 3.4. Tumor Microenvironment Heterogeneity

**Figure 5:**
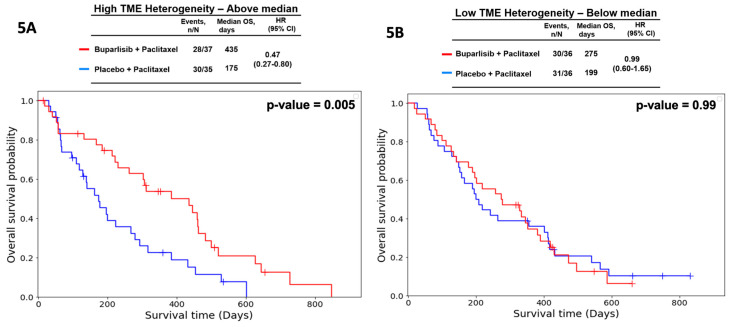
Tumor microenvironment cellular heterogeneity as a predictor of overall survival in buparlisib-treated patients. Kaplan–Meier curves of OS stratified by TME cellular heterogeneity, derived from H&E-based digital pathology. High-versus-low TME heterogeneity was defined according to the median cutoff. *Legend:* (**A**). High TME heterogeneity, (**B**). low TME heterogeneity.

We next assessed the association between TME cellular heterogeneity and clinical outcomes using the Shannon entropy index derived from AI-based H&E analysis. Among patients with high TME heterogeneity (above median), a significant treatment effect of buparlisib versus placebo was observed in patients with high TME heterogeneity (HR, 0.47 (95% CI, 0.27–0.80; *p* = 0.005)), whereas no between-arm difference was seen in patients with low TME heterogeneity (Figure 5A). In contrast, no between-arm difference was observed in patients with low TME heterogeneity (HR, 0.99 (95% CI, 0.60–1.65; *p* = 0.99)) (Figure 5B). These findings suggest that increased cellular and spatial complexity within the TME may enhance sensitivity to PI3K inhibition and serve as a predictive biomarker of buparlisib benefit, rather than reflecting general prognostic differences.

### 3.5. Granulocyte Enrichment in the TIM

**Figure 6:**
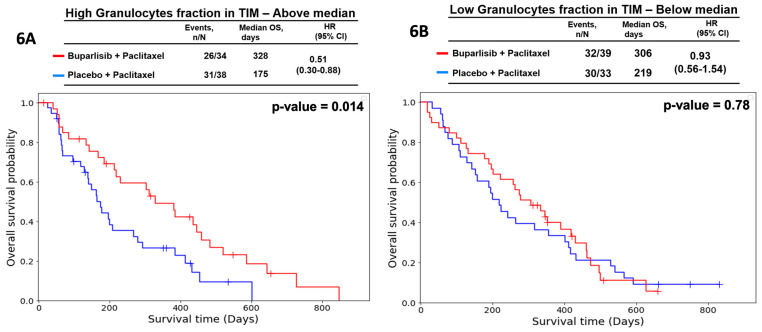
Granulocyte enrichment in tumor invasive margin as a predictor of overall survival in buparlisib-treated patients. Kaplan–Meier curves of OS stratified by granulocyte fraction in the TIM based on H&E-derived digital pathology. High-versus-low granulocyte fraction was defined according to the median cutoff. *Legend:* (**A**). High granulocyte fraction. (**B**). Low granulocyte fraction.

Among patients with high granulocyte infiltration in the TIM, as quantified by AI-based H&E analysis, buparlisib was associated with significantly improved OS compared to placebo (HR, 0.51 (95% CI, 0.30–0.88; *p* = 0.01)) (Figure 6A). In contrast, no between-arm difference was observed in patients with low granulocyte infiltration (HR, 0.93 (95% CI, 0.56–1.54; *p* = 0.78)) (Figure 6B). In a complementary proximity analysis based on the percentage of granulocytes within 50 µm of tumor cells in the tumor area, patients in the upper two thirds of this distribution had improved OS compared with the bottom third in the overall cohort (HR, 0.56 (95% CI, 0.39–0.82; *p* = 0.002)), an effect that was driven by the buparlisib + paclitaxel arm (HR, 0.32 (95% CI, 0.18–0.58; *p* < 0.001)), with no clear association in the placebo + paclitaxel arm (HR, 0.89 (95% CI, 0.52–1.45; *p* = 0.59)), supporting a predominantly predictive, rather than purely prognostic, effect.

## 4. Discussion

This study demonstrates that spatial biomarkers derived from AI-enabled analysis of H&E images were associated with differential therapeutic benefit from buparlisib in R/M HNSCC. Elevated tumor-TIL, high TME heterogeneity, and increased granulocyte infiltration in the TIM were each associated with improved OS benefit, suggesting a treatment-specific predictive effect rather than a purely prognostic one.

These findings build on prior BERIL-1 genomic and immunohistochemical profiling [8,9], while demonstrating the enhanced discriminatory capacity of H&E-based spatial metrics. Notably, AI-derived TIL quantification produced stronger HR and clearer outcome separation than conventional IHC scoring, highlighting the potential of digital pathology to refine immune-related biomarkers. While the CI for OS in high-TIL patients was wide (HR, 0.25 (95% CI, 0.01–0.64, *p* = 0.002)), the directionality and significance of the finding remain robust. However, formal comparative performance analyses between AI-derived spatial biomarkers and standard immunohistochemical or molecular biomarkers were beyond the scope of this exploratory study and warrant future evaluation. Consistent with the emerging literature on AI-based TIL quantification and spatial immune architecture in HNSCC, these findings further support the potential clinical utility of digital pathology-derived spatial biomarkers for treatment stratification and response prediction [20,21,22].

Similarly, high TME heterogeneity was associated with improved OS benefit from buparlisib treatment (HR, 0.47 (95% CI, 0.27–0.80)), underscoring the importance of spatial and cellular diversity in modulating response to PI3K inhibition. Elevated granulocyte infiltration in the TIM also correlated with improved buparlisib treatment effect (HR, 0.51 (95% CI, 0.30–0.88)), suggesting that granulocytes at the tumor–stromal interface may exert immunomodulatory effects that synergize with PI3K inhibition and chemotherapy. This potential interaction between innate immune infiltration and targeted PI3K blockade provides a mechanistic rationale for the relevance of these spatial biomarkers in the buparlisib plus paclitaxel setting [23,24]. Consistent with the emerging literature highlighting the predictive relevance of TME organization and intratumoral heterogeneity through AI-based spatial pathology approaches, these findings further support the biological and clinical relevance of spatial biomarkers in the buparlisib plus paclitaxel setting [25,26].

Although model performance was generally strong, moderate classification accuracy for certain phenotypes—particularly granulocytes and endothelial cells—remains an important limitation. Biomarker derivation relied on aggregate spatial metrics across large tissue regions rather than isolated single-cell predictions [27], which may partially mitigate the effect of individual classification errors.

This study also underscores the clinical utility of H&E-based spatial analysis in identifying candidates most likely to benefit from buparlisib plus paclitaxel. By capturing both established and spatially complex TME features, AI-driven models can detect immune infiltration, heterogeneity, and stromal interactions that are not easily discernible by conventional methods [28]. Although the phase 3 BURAN trial of buparlisib plus paclitaxel versus paclitaxel alone in PD-1/PD-L1-pretreated R/M HNSCC did not improve OS in the intent-to-treat population [29], H&E-derived spatial biomarkers remain cost-effective, interpretable, and scalable [30], supporting their relevance for biomarker-driven subgroup analyses within BURAN and future PI3K inhibitor studies. Importantly, H&E morphology does not permit reliable inference of specific immune subtypes or activation states that would require IHC or multiplex imaging.

This study has several limitations. It was a retrospective, post hoc analysis of a single phase II trial with a moderate sample size (*n* = 144), limiting power for subgroup and interaction effects. The moderate sample size also limited power for subgroup and biomarker-treatment interaction analyses, increasing the possibility of unstable effect estimates. All biomarker analyses were exploratory, with nominal two-sided *p*-values and no multiplicity adjustment, increasing the risk of type I error. A sex- and gender-based analysis was not performed because the H&E-derived biomarkers evaluated have limited known sex-specific biological variation in R/M HNSCC. While the deep learning pipeline performed well overall, detection accuracy for certain phenotypes (e.g., granulocytes, endothelial cells) was only moderate, and H&E-based classification remains imperfect. No independent, blinded validation set or prospectively locked thresholds were used, and variability in slide quality and digitization could have introduced noise or bias. Clinical confounding cannot be excluded in this paclitaxel combination setting, and generalizability beyond BERIL-1 is uncertain. Although this analysis was performed within the context of a randomized phase II clinical trial, the absence of an independent validation cohort limits generalizability of the findings and residual clinical confounding cannot be excluded. Subgroup analyses were exploratory and were not powered for definitive biomarker interaction testing. Accordingly, these results should be regarded as hypothesis-generating and require prospective validation. The ongoing BURAN phase III trial includes pre-specified biomarker definitions, centralized scanning, blinded algorithmic reads, and interaction testing to prospectively evaluate these spatial biomarkers.

## 5. Conclusions

AI-based spatial analysis of routine H&E slides offers a scalable, non-destructive approach to identify candidate predictive biomarkers of buparlisib efficacy in R/M HNSCC. These spatial features capture both established immune biomarkers, such as lymphocyte infiltration, and additional aspects of tumor–stromal architecture not detected by traditional assays. Their dual relevance underscores the biological plausibility of these findings in the context of PI3K inhibition plus chemotherapy and highlights their potential for patient selection. These findings support further evaluation of digital pathology approaches for biomarker-guided trial design and underscore the relevance of immune and stromal architecture in modulating PI3K inhibitor response. Prospective validation is warranted to confirm their clinical utility and guide personalized treatment strategies.

## Data Availability

The data presented in this study are available on request from the corresponding author. Access is restricted due to patient privacy concerns and institutional data-sharing agreements.

## Supplementary Materials

The following supporting information can be downloaded at: https://www.mdpi.com/article/10.3390/cancers18121887/s1, Supplemental Table S1: Quality control criteria of baseline FFPE tumor slides for whole-slide imaging; Supplemental Table S2: Baseline characteristics of patients with evaluable FFPE tumor samples; Supplemental Figure S1: Confusion matrices and class-specific performance metrics in the single-cell classification mode (A) and spatial segmentation model (B); Supplemental Figure S2: Distribution of HPV status across TIL Subgroups in Recurrent/Metastatic HNSCC tumors.
