# Supplementary material for: Spatial Biomarker Deep Learning Model Predicts Response to PI3K Inhibition in Head and Neck Cancer
Source: Cancers (Basel). 2026 Jun 10;18(12):1887. doi: 10.3390/cancers18121887 (PMC13297585; doi:10.3390/cancers18121887)
Supplement: Supplementary file 1 [file cancers-18-01887-s001.zip › Cancers_Buparlisib_digital_pathology_Supplemental_Figures S1 S2 Table S2_AD.pdf]

Online-Only Supplementary Material

Contents

- **Supplemental Table S1:** Quality control criteria of baseline FFPE tumor slides for whole-slide imaging

- **Supplemental Table S2:** Baseline characteristics of patients with evaluable FFPE tumor samples

- **Supplemental Figure S1:** Confusion matrices and class-specific performance metrics in the single-cell classification mode and spatial segmentation model

- **Supplemental Figure S2:** Distribution of HPV status across TIL subgroups in recurrent/metastatic HNSCC tumors

Supplemental Table S2. Baseline characteristics of patients with evaluable FFPE tumor samples

Parameters	Participants<br>N=144 (%)
**Age, years**	
**<65**	108 (75)
**≥65**	36 (25)
**Gender at birth**	
**Male**	121 (84)
**Female**	23 (16)
**Race**	
**Caucasian**	103 (72)
**Asian**	40 (28)
**Unknown**	1 (<1)
**Alcohol history, drinks/day**	
**< 1**	86 (60)
**> 1 and < 5**	45 (31)
**≥ 5**	8 (6)
**Unknown**	5 (4)
**Smoking status**	
**Former**	86 (60)
**Never**	32 (22)
**Current**	26 (18)
**HPV status**	
**p16 positive**	107 (74)
**p16 negative**	25 (17)
**Unknown**	12 (8)
**ECOG**	
**0**	0 (0)
**1**	94 (65)
**Unknown**	50 (35)
***PI3K* mutational status**	
**Altered**	16 (11)
**Non-altered**	96 (67)
**Unknown**	32 (22)
***TP53* mutational status**	
**Altered**	41 (28)
**Non-altered**	63 (43)
**Unknown**	40 (28)

Abbreviations: HPV : human papillomavirus, ECOG : Eastern Cooperative Oncology Group

S1A

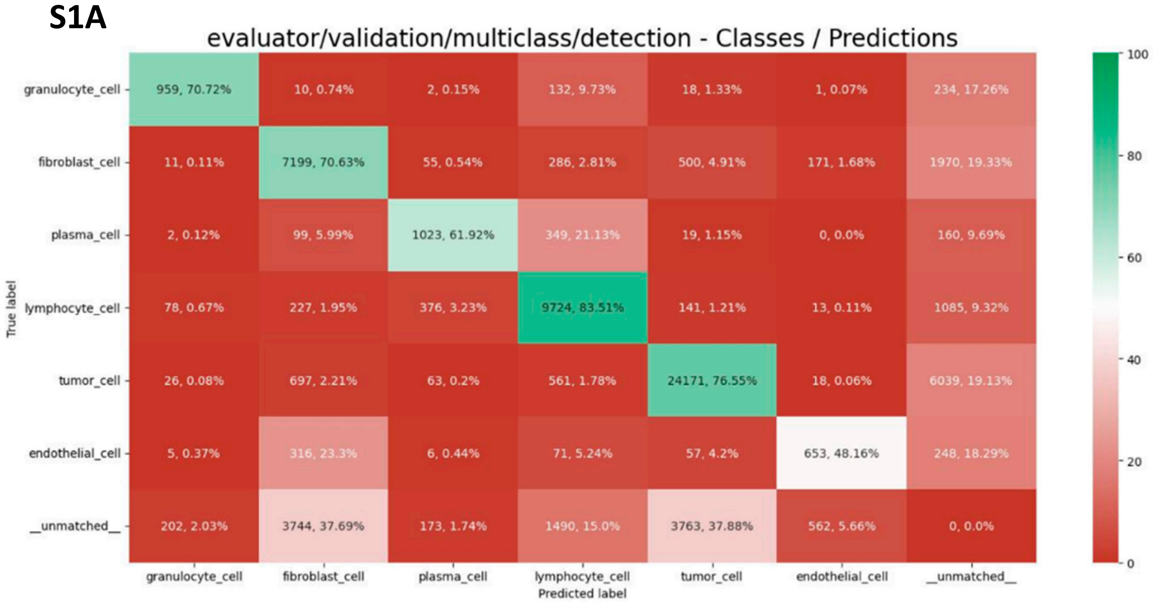

S1B

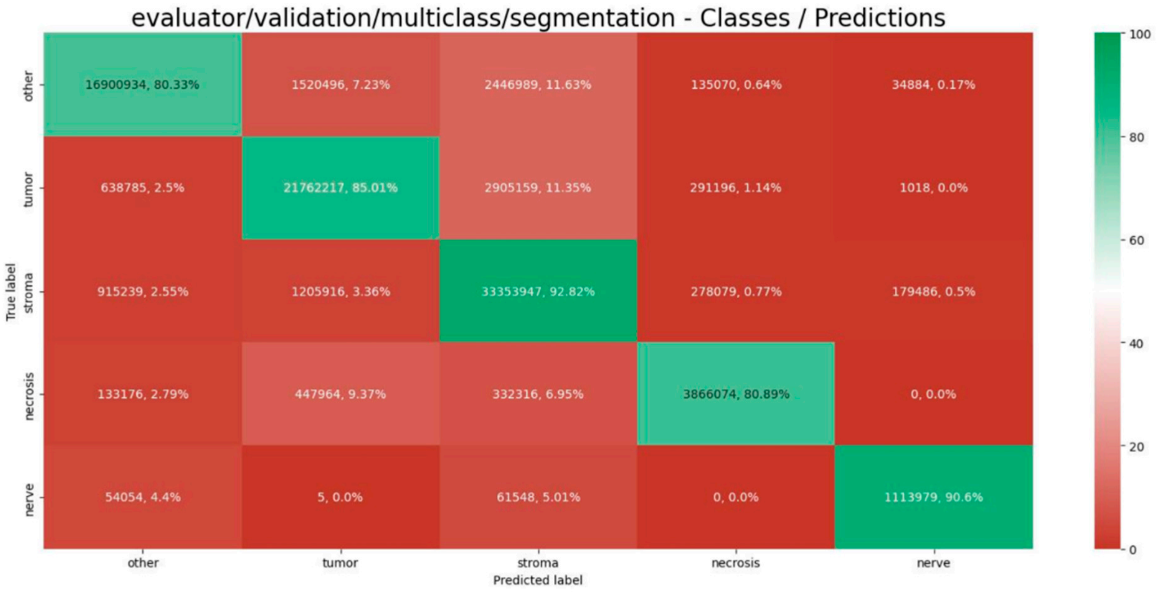

Supplemental Figure S1. Confusion matrices and class-specific performance metrics in the single-cell classification mode (S1A) and spatial segmentation model (S1B)

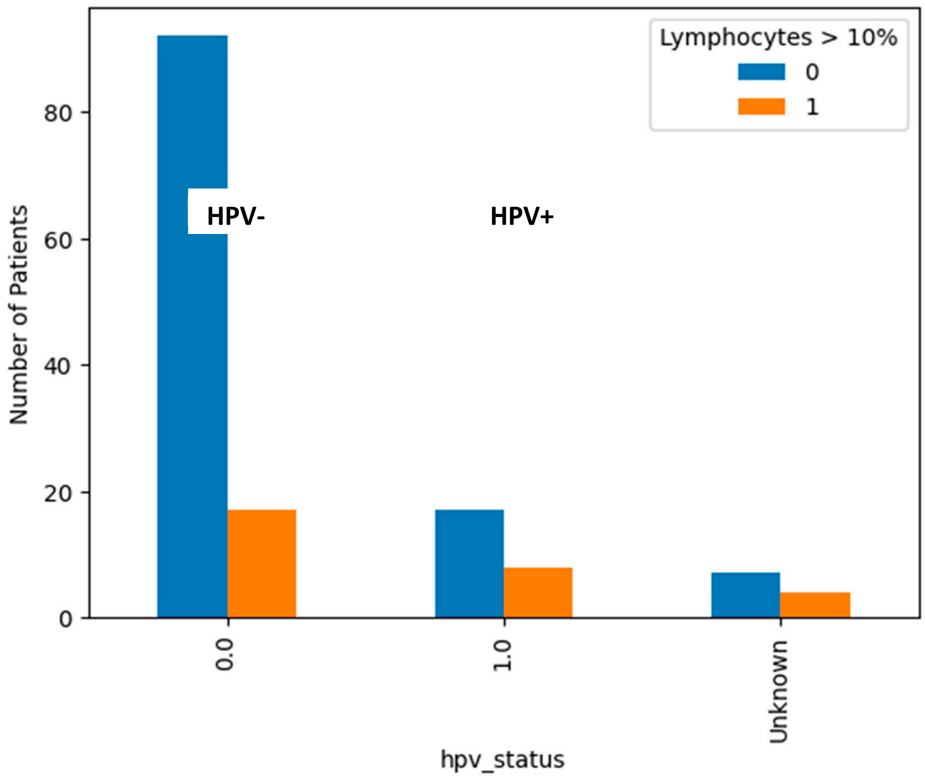

Supplemental Figure S2. Distribution of HPV status across TIL Subgroups in Recurrent/Metastatic HNSCC tumors

Legend: HPV status in patients with recurrent/metastatic head and neck squamous cell carcinoma was compared between those with ≥10% vs <10% tumor-infiltrating lymphocytes. HPV status was determined by p16 immunohistochemistry on baseline FFPE tumor samples, irrespective of tumor primary site.

Abbreviations: HPV: human papillomavirus; FFPE: Formalin-fixed paraffin embedded
